# Physico-Chemical Study of Curcumin and Its Application in O/W/O Multiple Emulsion

**DOI:** 10.3390/foods12071394

**Published:** 2023-03-25

**Authors:** Kristýna Opustilová, Barbora Lapčíková, Lubomír Lapčík, Shweta Gautam, Tomáš Valenta, Peng Li

**Affiliations:** 1 Department of Food Technology, Faculty of Technology, Tomas Bata University in Zlín, Nám. T. G. Masaryka 5555, 760 01 Zlín, Czech Republic; 2 Department of Physical Chemistry, Faculty of Science, Palacky University, 17. Listopadu 12, 771 46 Olomouc, Czech Republic

**Keywords:** curcumin, multiple emulsion, delivery system, emulsion stability, encapsulation, MDSC, HPLC

## Abstract

Curcuma is a world-renowned herb known for its immense health benefits. In this study, physicochemical analyses were performed on the curcumin standard sample and curcumin multiple emulsions. The emulsions were analysed for thermal and structural stability for 21 days. Confocal laser microscopy (CLSM) was performed in order to observe the emulsion encapsulation. Modulated differential scanning calorimetry (MDSC) and HPLC methods revealed a variety of curcuminoids (curcumin, demethoxycurcumin, bisdemethoxycurcumin, and cyclocurcumin) in the investigated curcumin standard. In addition, the MDSC method was found to be suitable and comparable to HPLC for determining the curcuminoid substances. The analysis of the curcumin release revealed a value of 0.18 w.% after 14 days as the equilibrium value. Furthermore, an increase in the sizes of the emulsions was observed at the end of the 21-day study. The emulsion stability index (ESI) was used to measure the stability of multiple emulsions. The ESI reached 55.8% between 7 and 21 days later. Nano droplets of the oil phase loaded with dispersed curcumin particles captured inside the water-based carboxymethylcellulose micelles were clearly observed by CLSM.

## 1. Introduction

Curcuma longa, or turmeric, is a member of the Zingiberaceae family, of the genus Curcuma. It is a well-known herb whose therapeutic and pharmacological properties are widely acknowledged. The plant is cultivated in the warm and humid climate of south Asian countries such as China, Taiwan, India, Singapore and Indonesia. Curcuma has a long history as a traditional medicine. It is widely used as a spice and colorant, and is a major ingredient of curry powder. Curcuma is harvested when the aerial part of this plant begins to senesce and curcuma rhizomes develop bright yellow to orange-yellow colour [1]. The rhizome is the primary source of the phytochemical substances in curcuminoids. Curcuminoids are polyphenolic compounds that provide the characteristic rhizome colour of Curcuma longa L. and account for about 2 to 9% of rhizome mass. The rhizome also contains bioactive and functional substances such as essential oils, acidic polysaccharides, sterols, metallic compounds, fatty acids, etc. Curcuminoid content in the raw rhizome depends on the cultivar variety, soil type, composition, and climate. Curcumin (1,7-bis(4-hydroxy-3-methoxyphenyl) hepta-1,6-diene-3,5-dione) is the principal curcuminoid that attracts the research niche [2]. Other curcuminoids include demethoxycurcumin, bisdemethoxycurcumin, and cyclocurcumin. Curcumin is known for its anti-cancer, antioxidant, anti-inflammatory, antimicrobial, and wound-healing properties [3,4,5]. It is also traditionally used for ailments such as cough, cold, fever, anaemia, bacterial and viral infections, minor skin infections such as eczema, and parasitic skin disease. The long list of health benefits has led to the development of novel products in the food, pharma and cosmetic industries. The food industry has a long history of using curcuma in various food formulations for its colour, taste, and health benefits [6]. According to European Union regulations, the limit of curcumin content in food supplements is 300 mg/kg, and for vegetable oils and fats, it is 5 mg/kg [7]. A higher intake of curcumin can induce adverse side effects in the human body, such as gastrointestinal distress, nausea, diarrhoea, and indigestion. It can also cause allergic reactions such as hives, rash, or itching [8,9].

Due to curcumins’ low bioavailability, rapid degradation (in acid, neutral, and alkaline conditions), and photochemical instability, different encapsulating approaches are utilized to enhance its utility [10]. One of them may be the use of liposomes. Liposomes are spherical vesicles containing one or more amphipathic phospholipid bilayers with an aqueous core inside and a hydrophobic layer on the surface. The most commonly used material for the formation of liposomes is highly purified lecithin, extracted from soybeans or egg yolks. The advantages of using liposomes are low toxicity (compared to the use of some polymer systems), biodegradability, biocompatibility, and solubilization of poorly soluble compounds [11]. Recent years have seen a trend in encapsulation via multiple emulsions. This can be simply defined as an “emulsion of emulsion”, meaning that the inner phase of the emulsion is itself an emulsion. There are two types of emulsions: water/oil/water (W/O/W) emulsions and reverse emulsion oil/water/oil (O/W/O). Although there are many methods for creating emulsions, the stability of these emulsions is still relatively low [12,13].

The encapsulation of curcumin in a multiple emulsion could effectively lead to protection from degradation and ensure controlled release in the digestive tract. Encapsulation protects the ingredients from unsuitable pH, oxygen, and heat conditions. The ingredients can be enclosed in various coating materials such as chitosan, gelatine, cellulose, modified starches, etc [14]. Natural sources of polysaccharides, such as citrus peels and sea weeds, also hold promising results in encapsulating the curcumin to enhance its bioactivity [15]. Such formulations can be used to produce products such as mouthwash [16], supplements [17], beverages, biscuits [18], milk [19], sausage [20], pasta, cheese [19], bread, and other daily household foods. It is pertinent to mention that there are some options already on the market in relation to commercial products. CurcuWin^®^ (OmniActive), LongVida^®^ (Ingenuity), NovaSol^®^ (CleanFoods), and Theracurmin^®^ (Natural Factors) are examples of turmeric extracts that are more bioavailable [21]. Furthermore, the encapsulated materials can be designed into functional, active packaging materials [22,23,24,25].

One of the most commonly sought methods of encapsulating curcumin is emulsification. Multiple emulsions are a complex system of O/W and W/O emulsions balanced by suitable emulsifiers. Furthermore, nanoscale multiple emulsions belong to a specific classification of emulsions, in which the mean droplet particle size ranges from 20 to 500 nm, compared to conventional emulsions (in which droplet particle diameters range from 100 nm to 100 μm). Nanoemulsions possess high kinetic stability due to their extremely small emulsion droplet sizes [26]. Multiple emulsions are usually formulated by a two-step process. The first step is the formation of the inner O/W or W/O single emulsion, followed by the dispersion of the emulsion in the outer phase through emulsification. The complex structure of multiple emulsions renders them thermodynamically unstable, resulting in the irreversible unwinding of the emulsion into the respective bulk phases [27].

The mechanism of emulsion instability includes, for example, diffusion of the inner oil phase into the outer oil phase due to the disruption of the separating aqueous phase and subsequent coalescence of droplets of the inner oil phase with the outer oil phase. This leads to a loss of encapsulation efficiency. However, this mechanism can also have a positive impact in terms of the controlled release of the encapsulated substances, yet this is complicated for multiple emulsions because of the dominance of the signal from the measurement of the outer oil globules and the limiting measurement of the inner droplets. Therefore, a procedure is used whereby the inner phase is first measured separately before forming the multiple emulsion. In addition to particle size, the amount of aqueous phase retained in the oil droplets is also important for the stability of the multiple emulsion and the efficiency of the encapsulation, which can be determined by differential scanning calorimetry (DSC). DSC provides information on changes in the size of inner water droplets and also determines the amount of water in the inner phases [28,29].

This study aimed to apply modern thermo-analytical methods of modulated differential scanning calorimetry (MDSC), followed by HPLC analysis of the model curcumin standard and verification of its possible encapsulation in the form of O/W/O multiple emulsions. Emulsion stability was followed by DSC over 21 days of storage and was quantified by the emulsion stability index (ESI) and the released curcumin content (w.%). It should be noted that the curcumin release kinetics in the simulated GI tract was not performed in this study.

## 2. Materials and Methods

In this study, a curcumin standard of 95 w.% total curcuminoid content was purchased from Alfa Aesar (USA) and stored at (4 ± 1) °C. Sunflower oil (ARO, MCC Trading International, Düsseldorf, Germany), soy granulated lecithin (Mogador, Otrokovice, Czechia), and carboxymethyl cellulose (CMC) of M_w_ = 250 kDa (Ashland, OR, USA) were purchased from local market. These products were stored in a dry and dark place at the ambient laboratory temperature of (25 ± 1) °C and relative humidity of 40%.

The chemicals and solvents used were of analytical grade: acetonitrile (HPLC super gradient, ≥99.9%) (Avantor Performance Materials, Gliwice, Poland), methanol (Chromasolv for HPLC, ≥99.9%) (Honeywell, Charlotte, NC, USA), and acetic acid glacial (≥99.9%) (Sigma Aldrich, Saint Louis, MO, USA). Distilled water (conductivity of 0.07 µS/cm) and ultrapure water were prepared on Aqua Osmotic 02 (AquaOsmotic, Tišnov, Czechia) and Aqua Max-Basic water purification systems (Young Lin Instrument, Anyang, Republic of Korea).

### 2.1. HPLC Analysis of Curcumin

For preparation of curcumin stock solutions (1.01 mg/mL), 10.1 mg of curcumin standard was dissolved in 10 mL of methanol (MET). The solution was then sonicated for 15 min in ultrasonic bath (type K2L, Kraintek, Hradec Králové, Czechia) at 10% bath power (at 35 kHz frequency). It was then cooled to the ambient laboratory temperature of 25 °C. Prior to the analysis, the solution was vigorously shaken, diluted with MET, and filtered on 0.45 µm microporous ceramic filter membrane. The testing solution of 100 µg/mL curcumin concentration was obtained. The standard curcumin curve was prepared by dilution of test solution with methanol in the following concentrations: 50, 20, 10, 5, and 1 µg/mL. HPLC analysis used was according to the modified method of Xiao and Sedlařík [30,31,32,33]. A liquid chromatograph Dionex Ultimate 3000 system (Thermo Scientific, Waltham, MA, USA) was used. Chromatographic separation was realized by a reversed phase Kinetex C-18 column (Phenomenex, Torrance, CA, USA) (150 × 4.6 mm; 2.6 µm) with 5 µL of the injected sample into the column; applied running time was 15 min. A constant flow rate of 1.0 mL/min was set and the column oven temperature was 40 °C. UV-VIS absorption was followed by Diode Array Detector (DAD) at a detection wavelength of 420 nm. Peak integration was performed by means of Chromeleon 7.2.10 (Thermo Scientific, USA) software. To achieve both high quality resolution and short elution time, optimal mobile phase composition was set as 4% acetic acid ultrapure water solution (*v/v*) (mobile phase A, phase ratio 30%) and 99.9% acetonitrile (ACN, mobile phase B, phase ratio 70%). Curcumin HPLC identification limits of detection and quantification were determined as well. Limit of detection (*LOD*) was calculated by the following equation:

(1) $$LOD=3.3\times \left(\sigma /S\right)$$

where *σ* represents the standard deviation of the response, and *S* the mean slope of the corresponding calibration curve.

Limit of quantification (*LOQ*) was calculated using the equation:

(2) $$LOQ=10\times \left(\sigma /S\right)$$

where *σ* is the standard deviation of the response, and *S* the mean slope of the corresponding calibration curve [6,34].

### 2.2. Preparation of Curcumin Multiple Emulsions (O/W/O)

The inner phase of the emulsion was prepared as follows: 3 g of granulated lecithin acting as an emulsifier was dissolved in 50 mL of distilled water. Curcumin was added to 15 mL sunflower oil to prepare a 1% solution (*w/w*), mixed thoroughly overnight, and sonicated in an ultra-sonic bath until completely dissolved. Subsequently, these two solutions were mixed for 10 min on a magnetic stirrer, followed by the addition of 20 mL (2.5 w.%) carboxymethylcellulose (CMC) solution as a stabilizer. It was then mixed in a high-speed blender (Philips Viva Collection blender 600 W HR 2170, The Netherlands) at a speed of 11500 rpm for 20 min at the laboratory temperature. The multiple emulsion was prepared by adding the inner phase dropwise into 15 mL sunflower oil with the application of high-speed blender. The optimal double emulsion system (O/W/O) had a phase ratio of 15/70/15 (mL), optimized by varying oil and water phase volumes and relevant contents of stabilizer and emulsifier based on our previous studies [35,36]. Samples were stored at a temperature of (4 ± 1) °C and relative humidity of (50 ± 5)%.

### 2.3. Dynamic Light Scattering (DLS) of Curcumin Multiple Emulsions

Particle size of the prepared emulsions was measured by dynamic light scattering (ZetaPlus Instrument, Brookhaven Instruments Corporation, Holtsville, NY, USA). Samples were diluted 10 times in distilled water. Measurement parameters were set as follows: refractive index of water 1.3330, refractive index of continuous oil phase 1.4340, laser wavelength 658 nm, and detection angle 90°. The inner phase of the emulsion was measured separately. Multiple emulsions were measured during a storage time of 21 days. Each measurement was repeated 10 times in 3 sets.

### 2.4. MDSC Analysis of Curcumin

Thermal properties of curcumin were analysed by modulated differential scanning calorimetry (MDSC) on DSC 250 instrument (TA Instruments, New Castle, DE, USA). The DSC instrument was calibrated by indium as a temperature standard. A total of (1.4 ± 0.1) mg of the sample was loaded into a non-hermetic aluminium pan. Measurements were set as follows: equilibrate to 140 °C, modulate temperature of 1 °C for 120 s, applied isothermal process for 10 min, and heating to 190 °C (heating rate of 2 °C/min) under nitrogen atmosphere (flow rate of 50 mL/min). An empty pan was used as the reference. Thermal events were characterized by detecting T_o_ (onset) and T_p_ (peak) temperatures. Enthalpy changes were defined as normalized enthalpies ΔH (J/g) and were calculated from the DSC peak areas [37].

### 2.5. UV-VIS Detection of Curcumin in Multiple Emulsions

Studied O/W/O emulsions were dissolved in methanol, shaken in vortex for 1 min followed by filtration through 0.45 μm membrane filter. Absorbance was measured at 420 nm wavelength on colorimeter Cecil CE 1021 (Cecil Instruments, Cambridge, UK) [38]. Curcumin released from the multiple emulsion was measured immediately after the emulsion preparation, and then regularly over 21 days. All measurements were repeated three times in triplicate. The device was calibrated using the curcumin calibration curve (420 nm); y = 0.2959x − 0.0101. 

### 2.6. Determination of Emulsion Stability by DSC

Emulsion stability was measured after 0, 7, 14, and 21 days of storage on DSC 250 (TA Instruments, USA) by the procedure of Dalmazzone et al. [39]. Emulsion samples were taken from different layers of the separated samples (top, middle and bottom). The DSC instrument was calibrated by indium as a temperature standard. Specific weights of O/W/O emulsion of (10.0 ± 0.5) mg were filled into hermetically sealed aluminium pans with pinhole lids. The experimental conditions were set as follows: cooling from 25 °C to −50 °C (at 2.5 °C/min cooling rate), keeping isothermal process for 1 min, followed by heating from −50 °C to 30 °C (at 5 °C/min heating rate). All experiments were performed under nitrogen atmosphere (nitrogen flow rate of 50 mL/min). An empty pan was used as a reference. Thermal events were evaluated by detection of T_o_ (onset) and T_p_ (peak) temperatures. Enthalpy change was defined as normalized Δ*H* (J/g) and calculated from DSC peak area [39,40]. Freezable water content (*W_fs_*) was expressed as the ratio of the emulsion melting enthalpy and pure water endothermic enthalpy:

(3) $$W_{fs}=\frac{\Delta H_{s}}{\Delta H_{H_{2}0}}\times 100\%$$

where *W_fs_* is free and bounded freezable water content, Δ*H*_s_ is total enthalpy of freezable water and ∆*H_H_*_2_*_O_* = 333.5 J/g is melting enthalpy of pure water [41].

### 2.7. Determination of Emulsion Stability by ESI

The sample of multiple O/W/O emulsion was centrifuged at 6000 rpm for 20 min on an EBA 21 centrifuge (Andreas Hettich, Tuttlingen, Germany). Subsequently, the emulsion was placed into measuring cylinder, and the separation of the oil (top) and aqueous (bottom) phases was monitored over time at 4 °C. Emulsion stability index (*ESI*) was calculated as follows [42]:

(4) $$ESI=\left(1-\frac{volume\;of\;bottom\;phase}{total\;volume\;of\;emulsion}\right)\times 100\%$$

*ESI* was determined over the period of 21 days of storage.

### 2.8. Confocal Laser Scanning Microscopy (CLSM)

For visualization of the studied O/W/O double emulsion system, a Zeiss LSM780 confocal microscope (Germany) equipped with a Plan-Apochromat 63 ×/1.4 Oil DIC M27 objective was used. The image size was set to 134.95 µm × 134.95 µm, and the scanning on the *Z*-axis was every 0.7 µm. Recorded images were analysed using ZEN software (Zeiss, Germany). Both images were captured after 7 days of preparation. Images represent sample areas followed at 467 nm and at 580 nm.

### 2.9. Statistical Analysis

Obtained experimental data were analysed by one-way analysis of variance ANOVA with Duncan’s multiple range test. Differences in the mean values among statistical groups were tested at a significance level of α ≤ 0.05. SigmaPlot ver. 12.5 (Systat Software, San Jose, CA, USA) was used for data analysis.

## 3. Results

### 3.1. HPLC Analysis

**Figure 1:**
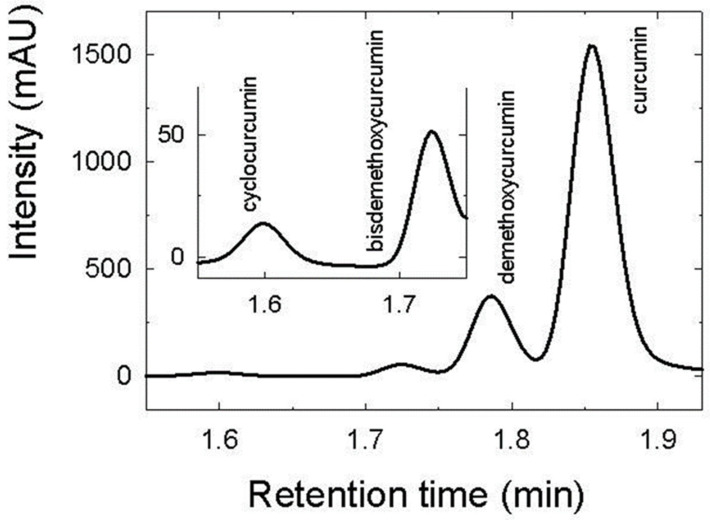
HPLC chromatogram of curcumin standard (50 µg/mL). Inset: expanded chromatogram retention time interval of 1.55 to 1.75 min.

Results of the HPLC analysis are shown in Figure 1. Here, four peaks were detected reflecting varying content of curcuminoids present in the samples. Curcumin was identified as the main curcuminoid by the occurrence of the relevant elution peak observed at retention time (1.853 ± 0.004) min. This was in agreement with the results reported by Syed et al. [6]. The authors [6] assigned the highest peak to curcumin, and smaller peaks to other curcuminoids. In the present study, the curcumin peak was preceded by a minor peak of cyclocurcumin at (1.600 ± 0.002) min retention time, and lower peaks of bisdemethoxycurcumin at (1.723 ± 0.004) min and demethoxycurcumin at (1.787 ± 0.001) min. The HPLC measurements were quantified by the obtained standard curve (y = 1.0587x − 0.3912). The limit of detection (LOD) of curcumin was 11.08 ng/mL, and the limit of quantification (*LOQ*) was 33.57 ng/mL. 

The curcumin content observed for 50 μg/mL sample dose was (41.02 ± 0.08) µg/mL (82.02 w.% of the total detected curcuminoids). The concentration of other curcuminoids found was: cyclocurcumin (0.55 ± 0.02) µg/mL (1.11 w.%), bisdemethoxycurcumin (0.95 ± 0.02) µg/mL (1.90 w.%), and demethoxycurcumin (7.49 ± 0.01) µg/mL (14.97 w.%). The obtained ratio was similar to those reported by Yixuan [43]. 

### 3.2. MDSC Analysis

**Figure 2:**
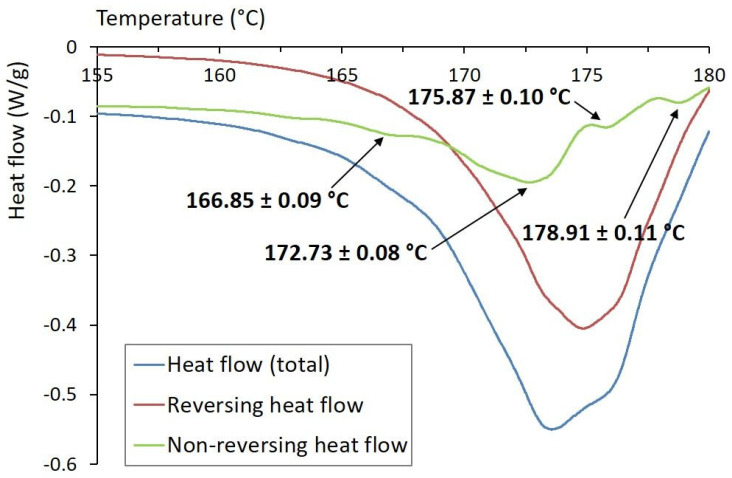
MDSC total heat flow pattern, and its reversing and non-reversing components, of studied curcumin powder.

Results of the MDSC analysis are shown in Figure 2. There were clearly recognized reversing and non-reversing heat flow patterns, indicating specific curcuminoids’ composition. Total heat flow represented the sum of the reversing and non-reversing heat flows. Reversing processes were represented by melting heat capacity, whereas the non-reversing processes were related to the kinetic components of samples’ melting [44]. For the reversible events, one endothermic peak with onset temperature T_o_ = (168.92 ± 0.11) °C, peak temperature T_p_ = (174.83 ± 0.07) °C, and normalized enthalpy of fusion Δ*H* = (80.83 ± 0.15) J/g was observed. It was attributed to the melting of curcumin crystals. The obtained results were consistent with the melting peaks reported by Sun et al. [44] and Sayyar et al. [45], who identified the melting point of curcumin at 175 °C. Four endothermic peaks were detected on the non-reversing heat flow curve, as shown in Figure 2, and were ascribed to different curcuminoids. A minor peak detected at T_o_ = (165.64 ± 0.13) °C, T_p_ = (166.85 ± 0.09) °C, and characterized by a small enthalpy change Δ*H* = (0.216 ± 0.021) J/g, was associated with cyclocurcumin as a minor curcuminoid substance [20]. The largest peak observed at T_o_ = (168.90 ± 0.11) °C, T_p_ = (172.73 ± 0.08) °C, with enthalpy of fusion of Δ*H* = (8.50 ± 0.14) J/g, was associated with curcumin. The other two peaks, detected at T_o_ = (175.26 ± 0.12) °C, T_p_ = (175.87 ± 0.10) °C, with Δ*H* = (0.52 ± 0.04) J/g and at T_o_ = (177.99 ± 0.08) °C, T_p_ = (178.91 ± 0.11) °C, with Δ*H* = (0.42 ± 0.04) J/g, were ascribed to demethoxycurcumin and bisdemethoxycurcumin, respectively. Based on the above data, it can be assumed that both MDSC and HPLC techniques have the capacity to provide comparable qualitative and quantitative results.

### 3.3. Curcumin Release Pattern Emulsions’ Stability

Curcumin release was followed by UV-VIS spectrophotometry. The curcumin release was facilitated using methanol. It was expressed as a percentage of the original curcumin present within the emulsion system. The initial curcumin content was about 0.11 w.% (determined immediately after preparation). After 7 days of storage, the curcumin content increased to 0.17 w.% and 0.18 w.% after 14 and 21 days, respectively. After 14 days, the released content reached an equilibrium of 0.18 w.%. Such behaviour can be characterized as sustained release [46].

Due to the fact that the colloidal stability of emulsions is influenced by the particle size of the individual droplets, there were DLS measurements performed, which indicated an inner phase particle diameter of (167 ± 5) nm (immediately after the O/W system preparation). The O/W phase exhibited colloidal stability over 21 days. On the contrary, the stability of the O/W/O emulsion was only maintained for 7 days. The mean diameter of the multiple emulsion was (392.5 ± 11.5) nm (immediately after preparation). After 7 days, it increased to (663.6 ± 7.7) nm, and to (641.67 ± 13.9) nm after 21 days. With the observed increasing particle size, there was observed coalescence followed by creaming [47]. 

**Figure 3:**
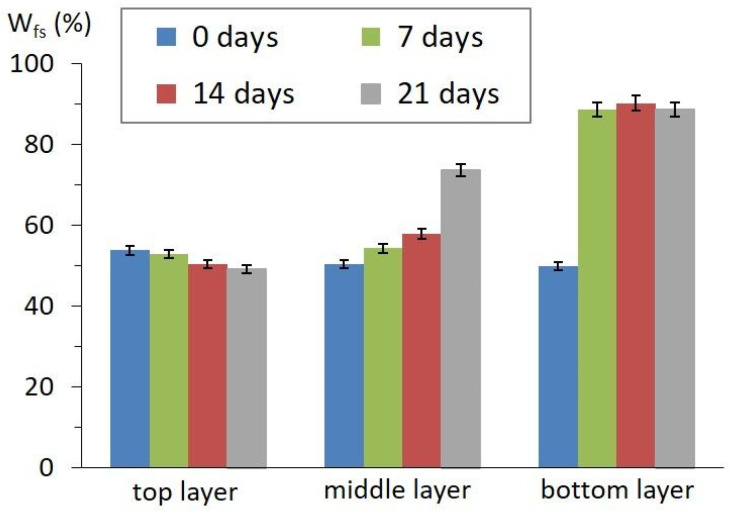
Freezable water content (*W_fs_*) trend during storage. (Data represented as arithmetic means of three measurements with standard deviation).

The stability of multiple emulsions was characterized by the emulsion stability index (*ESI*). After 7 to 21 days, ESI reached 55.8%. Emulsion stability was also evaluated by DSC (Figure 3). There was an observed increase in the *W_fs_*, from 0.5 (immediately after preparation) to 0.74 and 0.89 (after 21 days) for the middle and bottom layers, respectively. This increase in *W_fs_* can be associated with the degradation of the inner phase accompanied by water separation, as indicated by the increased ratio of *W_fs_* for the bottom layers. Conversely, there was a negligible decrease in *W_fs_* (0.54 to 0.49) for the top layers, due to emulsion creaming. 

### 3.4. Confocal Laser Scanning Microscopy

**Figure 4:**
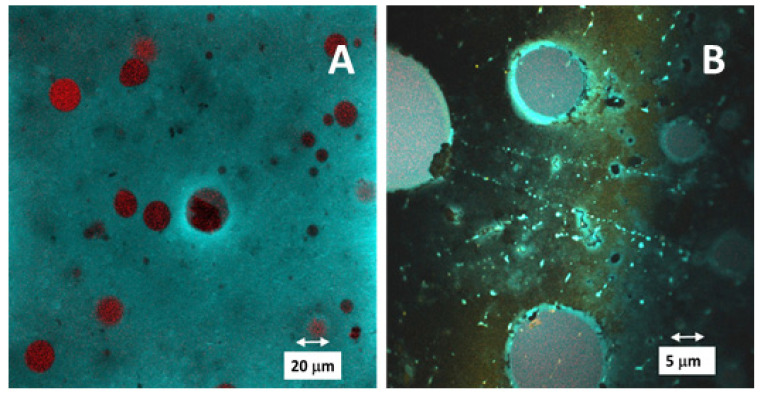
CLSM micrographs of O/W/O emulsion captured 7 days after preparation. Images represent sample areas followed at 467 nm (**A**) and at 580 nm (**B**). The excitation wavelength was 405 nm, with emission filters set 415–500 nm, and 540–600 nm.

Results of the CLSM microscopy of studied O/W/O emulsions are shown in Figure 4. It was confirmed that nano droplets of the oil phase, loaded with dispersed curcumin particles, were captured inside the water-based carboxymethylcellulose micelles (see Figure 4A) stabilized by lecithin (see Figure 4B). Observed CLSM images represented the O/W/O emulsion system. Unbound curcumin nanoparticles were dispersed in the surrounding oil phase.

## 4. Conclusions

The results of the study presented confirmed successful encapsulation of the curcumin in O/W/O emulsion. It was concluded that the prepared O/W/O curcumin nanoemulsion could potentially serve as a vehicle matrix for curcumin food delivery systems. Different curcuminoids in the studied curcumin powder were detected by the MDSC and HPLC techniques. Furthermore, it was found that the MDSC technique may be used as a suitable tool for the determination of various curcuminoid substances occurring in curcumin samples. Multiple emulsions were prepared and analysed in order to study the particle size distribution, thermal stability, curcumin release pattern, emulsion stability and encapsulation structure. Curcumin release analysis revealed a value of 0.18 w.% as the equilibrium value after 14 days. It was also found that the sizes of the emulsions increased considerably by the end of study. This behaviour was attributed to coalescence, followed by creaming. The emulsion stability analysis revealed the degradation of the inner phase of the bottom and middle layers of the emulsion. For the characterization of the multiple emulsions, CLSM microscopy was used, indicating the creation of the approximately 20 mm particles of the CMC micelles stabilized by lecithin, loaded with oil-phase-dispersed nano curcumin particles. With respect to future trends, the functional properties and release kinetics of curcumin from the emulsion structure can be an attractive research aspect. Moreover, this study also paves the way for studies related to the stability kinetics of O/W/O multiple emulsions with regards to a gastro-intestinal tract model.

## Data Availability

The datasets generated in this study are available on request to the corresponding author.
